# Associations of lifetime cumulative estrogen exposure with lifecourse social exposures, cognitive decline, and dementia risk among postmenopausal White, Black, and Latina women

**DOI:** 10.1093/geronb/gbag001

**Published:** 2026-01-10

**Authors:** Justina F Avila-Rieger, Benjamin Huber, Sarah E Tom, Whitney R Robinson, Tanisha G Hill-Jarrett, Mateo P Farina, Timothy J Hohman, Nicole Schupf, Adam M Brickman, Richard P Mayeux, Jennifer J Manly

**Affiliations:** Department of Neurology, College of Physicians and Surgeons, Columbia University, New York, New York, United States; Gertrude H. Sergievsky Center, College of Physicians and Surgeons, Columbia University, New York, New York, United States; Department of Neurology, College of Physicians and Surgeons, Columbia University, New York, New York, United States; Department of Neurology, College of Physicians and Surgeons, Columbia University, New York, New York, United States; Department of Epidemiology, Columbia University, New York, New York, United States; Division of Women’s Community and Population Health, Department of Obstetrics and Gynecology, Duke University School of Medicine, Durham, North Carolina, United States; Department of Neurology, Memory and Aging Center, University of California, San Francisco, San Francisco, California, United States; Global Brain Health Institute, University of California San Francisco, San Francisco, California, United States, and Trinity College Dublin, Ireland; Department of Human Development and Family Sciences, University of Texas at Austin, Austin, Texas, United States; Vanderbilt Memory & Alzheimer’s Center, Vanderbilt University Medical Center, Nashville, Tennessee, United States; Department of Neurology, College of Physicians and Surgeons, Columbia University, New York, New York, United States; Gertrude H. Sergievsky Center, College of Physicians and Surgeons, Columbia University, New York, New York, United States; Department of Neurology, College of Physicians and Surgeons, Columbia University, New York, New York, United States; Gertrude H. Sergievsky Center, College of Physicians and Surgeons, Columbia University, New York, New York, United States; Department of Neurology, College of Physicians and Surgeons, Columbia University, New York, New York, United States; Gertrude H. Sergievsky Center, College of Physicians and Surgeons, Columbia University, New York, New York, United States; Department of Neurology, College of Physicians and Surgeons, Columbia University, New York, New York, United States; Gertrude H. Sergievsky Center, College of Physicians and Surgeons, Columbia University, New York, New York, United States

**Keywords:** Reproductive health, Alzheimer’s disease, Social determinants, Cognitive health

## Abstract

**Objectives:**

Greater lifetime exposure to estrogen may protect women from cognitive decline and dementia later in life. Gender-related social factors also influence women’s cognitive outcomes; however, little is known about how these biological and social influences work together. We examined the extent to which cumulative estrogen exposure and lifecourse social exposures jointly influence late-life memory trajectories and dementia risk among a community-based sample of White, Black, and Latina women.

**Methods:**

Participants were 3,688 postmenopausal women in the Washington Heights-Inwood Columbia Aging Project. Lifetime cumulative estrogen exposure was estimated based on age at menarche and menopause, breastfeeding duration, and hormone replacement therapy use. Lifecourse social factors included birth cohort, childhood socioeconomic status (SES), educational and occupational attainment, and later-life income. Multiple-group growth models and Cox regression models were estimated across racial and ethnic groups.

**Results:**

Greater lifetime estrogen exposure was independently associated with higher baseline memory performance among Black and Latinx women, slower memory decline among White women, and lower dementia risk among Latinx women. Later birth year and higher lifecourse SES were associated with greater lifetime estrogen exposure, with associations varying in magnitude across racial and ethnic groups. Associations between lifecourse SES and each cognitive outcome were partially mediated by estrogen exposure indicators.

**Discussion:**

Cumulative estrogen exposure is socially patterned. We found that lifecourse social factors and estrogen exposure synergistically contribute to women’s late-life cognitive health outcomes. Understanding how sex-linked biology and gender-related social forces intertwine is essential for developing interventions to decrease dementia risk among women.

Alzheimer’s disease (AD) disproportionately affects women (“2025 Alzheimer’s Disease Facts and Figures,” 2025), yet the sex-linked biological and social determinants of women’s AD risk are not well understood. The sex-steroid hormone estradiol (E2) is thought to play an important role in shaping AD risk among women (Snyder et al., 2016). Animal and cellular models consistently show the neuroprotective effects of E2 through several mechanisms, including increasing hippocampal dendritic spine density (Cooke & Woolley, 2005), increasing choline acetyltransferase activity in the hippocampus (Aenlle et al., 2009), reducing the aggregation of amyloid-beta neurotoxicity (Nilsen et al., 2006), and preventing mitochondrial damage (Yao & Brinton, 2012). It is hypothesized that depletion of E2 with menopause increases susceptibility to AD-related brain changes (Kunzler et al., 2015; Rahman et al., 2020). However, increasing evidence suggests that greater exposure to E2 earlier in life may provide long-lasting cognitive protection (Jett et al., 2022). Understanding how estrogen exposure influences AD risk across the lifecourse can lead to the identification of critical windows for intervention at each reproductive stage in a woman’s life.

Various aspects of reproductive history determine cumulative estrogen exposure. For example, estrogen levels increase at menarche and during menopause. A longer length of time between menarche and menopause (i.e., length of reproductive period) is often associated with increased endogenous estrogen exposure (Hesson, 2012). Estrogen levels rise to 300-fold throughout pregnancy and fall 100–1000-fold postnatally, often due to breastfeeding (Schock et al., 2016). Longer duration of breastfeeding, which influences the number of menstrual cycles within a woman’s reproductive years, also reduces cumulative exposure to endogenous estrogen (Bernstein, 2002). Parous women may also have shorter menstrual cycles and lower levels of E2 than nulliparous women (Smith et al., 1999). Moreover, the clinical response to HRT may depend on its interaction with these reproductive history factors (Rasgon et al., 2005).

Several studies have combined these estrogen-altering reproductive features into an index of total lifetime duration of estrogen exposure (i.e., cumulative estrogen exposure index [CEEI]) (Rasgon et al., 2005; Smith et al., 1999). Higher CEEI scores (i.e., higher levels of cumulative estrogen exposure) have been associated with higher scores on memory tests (Hesson, 2012; Matyi et al., 2019; Smith et al., 1999) and a lower risk for AD (Fox et al., 2013; Prince et al., 2018). The CEEI is more consistently related to cognitive outcomes than individual markers (Fox et al., 2013; Rasgon et al., 2005; Smith et al., 1999), suggesting that combining the effects of endogenous and exogenous estrogen markers may be a beneficial approach to understanding the influence of estrogen on the brain and cognitive outcomes.

Women’s AD risk is also influenced by several lifecourse social exposures, including time and place of birth, early life socioeconomic status (SES), and adult SES (i.e., educational and occupational attainment, income) (Mielke et al., 2022). Interestingly, these lifecourse factors are also associated with indicators of cumulative estrogen exposure. In the United States, later birth cohort (Nabhan et al., 2022) and having high childhood SES (Hiatt et al., 2021) are associated with an earlier age at menarche. Higher adult SES is associated with later age at natural menopause (Schoenaker et al., 2014), lower parity (Patel et al., 2022), and a higher likelihood and longer duration of HRT use (Iyer & Manson, 2024). Associations between lifecourse social exposures and reproductive history are stronger among non-Latinx White women compared with other racial and ethnic groups (Chumlea et al., 2003; Hiatt et al., 2021; Krieger et al., 2015). Given this evidence, reproductive health outcomes may be on the causal path linking social exposures to AD risk. However, very little is known about the synergistic contributions of cumulative estrogen exposure and lifecourse social factors on late-life cognitive health outcomes.

The goal of the present study was to determine the extent to which cumulative estrogen exposure and lifecourse social exposures jointly influence memory trajectories and dementia risk among a community-based sample of White, Black, and Latinx postmenopausal women. It was hypothesized that greater cumulative estrogen exposure would be independently associated with higher baseline memory performance, slower memory decline, and a lower dementia risk across racial and ethnic groups. We expected that later birth year and higher childhood and adult SES would be associated with higher levels of cumulative estrogen exposure, with stronger associations present among White women compared with Black and Latinx women. We also hypothesized that associations of lifecourse social exposures with memory trajectories and dementia risk would be, at least, partially mediated by cumulative estrogen exposure.

## Method

### Participants

We used data from the Washington Heights-Inwood Columbia Aging Project (WHICAP), an ongoing study of community-dwelling Medicare recipients 65 years and older residing in northern Manhattan (Tang et al., 2001). Recruitment occurred in three waves: 1992 (*N* = 2,126), 1999 (*N* = 2,180), and 2009 (*N* = 2,301). Participants completed a baseline assessment and were followed up at 18 to 24-month intervals for up to 25 years. During each session, participants were administered a neuropsychological battery and asked about their life-course social history, general health, functional ability, and medical history. This study was approved by the Institutional Review Boards at Columbia University. Written informed consent was obtained.

The total WHICAP sample included 6,607 participants. We excluded participants who self-identified as men (*n* = 2,031) and participants who reported a primary race/ethnicity other than Latinx, non-Latinx White, or non-Latinx Black (*n* = 59). Participants who did not complete at least one full neuropsychological battery (*n* = 354) and those who met criteria for dementia at baseline (*n* = 475) were also excluded from the current analyses. The remaining sample (*n* = 3,688) included 840 White (23%), 1,177 Black (32%), and 1,671 Latinx (45%) women.

### Measures

#### Estrogen exposure variables

Participants were asked a series of questions regarding their reproductive health history, including age at menarche, age at menopause, reason for menopause (e.g., natural, surgical, chemotherapy/radiation treatment), history of reproductive surgery (e.g., bilateral oophorectomy, hysterectomy), number of pregnancies and live births, total number of months spent breastfeeding, hormone replacement therapy (HRT) use and duration (months/years). Our primary exposure, lifetime cumulative estrogen exposure, was estimated based on previous work in this area (Fox et al., 2013): cumulative estrogen exposure index (CEEI) = (reproductive span − years breastfed) + years of HRT use. Reproductive span was calculated as age at menopause minus age at menarche. To account for changes in pregnancy-associated ovulatory cycles, breastfeeding duration was defined as 1.5 months per live birth for parous women who did not breastfeed and 0.25 months per pregnancy that did not result in a live birth (Fox et al., 2013). The duration for nulliparous women who reported no pregnancies was zero. Duration of HRT use was defined as years of HRT use before or after menopause.

#### Lifecourse social exposures and experiences

Self-reported race and ethnicity were classified based on the 1990 U.S. Census guidelines. Participants were first asked whether they were Hispanic or Latino and then asked to classify themselves racially as White, Black, Asian, American Indian, Pacific Islander, or other. Birth cohort was measured continuously by centering the birth year at 1926 and dividing by 5. Each participant’s childhood socioeconomic status (CSES) factor score was derived through factor analysis based on their parental years of education, occupation, and number of siblings. The highest completed grade of school was used to measure years of educational attainment and centered at 11 years. Self-reported monthly household income at baseline was characterized into low (≤ $750 per month), medium ($751–$1,750), and high (≥ $1,751) income categories. Self-reported highest occupational status during the lifetime was measured using four categories: unskilled/semiskilled, homemaker, skilled/clerical, and managerial/professional.

#### Covariates

Baseline age was centered at the sample mean (76 years). APOE-ε4 status was defined as either positive (presence of at least one ε4 allele) or negative (absence of ε4 allele). BMI at baseline was included as a continuous variable.

#### Outcomes

Memory was assessed by the immediate, delayed, and recognition trials from the Selective Reminding Test (SRT) (Buschke & Fuld, 1974). Each variable was converted to standardized scores using the means and standard deviations from the entire WHICAP sample at their baseline visit. Composite scores were computed by averaging the standardized scores on each occasion.

Diagnosis of all-cause dementia was determined via consensus case conference based on neurological, neuropsychological, functional, medical, and psychiatric data collected from participants and/or informants and followed standard research criteria for all-cause dementia. Follow-up diagnoses were made blind to prior diagnoses.

### Statistical analysis

Supplementary Table 1 (see online supplementary material) shows the proportion of missing data for each of our key variables. Data are missing at random (MAR) and are completely predicted by the WHICAP recruitment cohort. To avoid the loss of power and potential bias associated with complete-case analysis, we performed multiple imputation by chained equations (see Supplementary Material for detailed information on imputation methods and diagnostics). Bayesian methods were used for all analyses, which are more robust than frequentist estimators when imputing a large amount of missing data (Linero & Daniels, 2018).

Memory trajectories were estimated via latent growth modeling, with time parameterized as years from participants’ initial visit. Joint modeling, which combines a latent growth model with a survival model, was used to account for the influence of differential attrition due to death on memory trajectories. We also included a retest spline to account for practice effects. Dementia risk was estimated using Cox regression models.

*Model 1* examined the independent association of the estrogen exposure index (EEI) on baseline memory/memory decline (latent growth model) and dementia risk (Cox regression), adjusting for age at baseline, age squared, APOE-ε4 status, and BMI at baseline. *Model 2* examined associations between the EEI and each lifecourse social variable—birth year, childhood SES, educational attainment, monthly income, and occupational attainment. *Model 3* combines Models 1 and 2, incorporating generalized structural equation modeling (Muthen & Muthen, 2017) and mediation analyses, with exposure–mediator interactions (VanderWeele, 2016), to examine the pathways linking the lifecourse social exposures to each cognitive outcome via the EEI. The total effect (TE) of lifecourse social factors on an outcome was decomposed into its components: the pure natural direct effect (PNDE; i.e., portion of the TE not mediated through the EEI) and the total natural indirect effect (TNIE; the mediated effect through the EEI). The TNIE is further decomposed into the pure natural indirect effect (PNIE; i.e., the indirect effect solely due to the mediated pathway) and the mediated interaction (INTmed; i.e., the portion of the TNIE that results from the exposure–mediator interaction). Mediation analyses were estimated using Bayes, and weakly informative prior distributions were specified for all path coefficients. Models 1–3 were reestimated for each of the reproductive health variables that comprised the EEI (i.e., age at menarche, age at menopause, reproductive span, duration of HRT use, duration of breastfed).

Finally, Multiple-group modeling examined racial and ethnic differences in estimated associations from each model. We estimated separate known-class mixture models, with racial and ethnic groups as the known-class grouping variable. This known grouping variable is incorporated into these models as a moderator variable, allowing model parameters to vary as a function of membership in the identified groups. Racial and ethnic differences in model parameters were tested using the “Model Constraint” option in Mplus version 8.6 (Muthen & Muthen, 2017).

Both *p*-values and confidence intervals were used to determine statistical significance. A *p*-value of .01 (i.e., 99% confidence interval) was used for multiple group comparisons to decrease the likelihood of type I error. *p*-values of .05 or 95% confidence intervals were used for analyses across the entire sample.

## Results

### Participant characteristics

Participant characteristics from imputed data are presented in Table 1 (non-imputed sample statistics are available in Supplementary Table 1). Approximately 40% of the White women were European-born, whereas the Latinx participants were primarily emigrants from the Caribbean (Puerto Rico, Dominican Republic, and Cuba). Most of the Black women (55%) were born in the U.S. South. Compared with Black and White women, Latinx women were younger at their baseline visit, born in a later birth cohort, had lower childhood SES and educational attainment, and were more likely to have low monthly income and be a homemaker or in an unskilled occupation most of their lives. Compared with Black and Latinx women, White women had the highest childhood SES and educational attainment, were more likely to have a high monthly income, and to be in a managerial or professional occupation. Lifetime cumulative estrogen exposure was highest among White women, followed by Black women, and then Latinx women, who had the lowest cumulative estrogen exposure. Latinx women gave birth to more children and breastfed for a longer duration compared with Black and White women. White women were more likely to use HRT and for a longer duration than their Black and Latinx counterparts. Black women had the lowest average age at menarche, age at menopause, and the shortest reproductive span.

**Table 1.** Sample characteristics from imputed data.

Characteristic	Full sample	White women	Black women	Latinx women
***N***	3,688	840	1,177	1,671
**Age at baseline (in years)**	75.9 (6.6)	76.4 (7.1)	76.1 (6.6)	75.6 (6.2)
**Birth year**	1,927 (11)	1,926 (11)	1,927 (11)	1,928 (10)
**Birthplace, % U.S. born**	45%	60%	92%	3%
**APOE-ε4 status, % positive**	27%	22%	34%	25%
**Body mass index**	28.5 (6.7)	26.5 (5.8)	29.3 (7.5)	29.0 (5.6)
**Childhood socioeconomic status**	0.03 (0.9)	0.50 (0.9)	0.12 (0.8)	−0.28 (0.9)
**Educational attainment**	10.1 (4.9)	13.8 (3.5)	11.8 (3.6)	7.0 (4.4)
**Income**				
** Low**	41%	15%	32%	60%
** Medium**	36%	33%	40%	34%
** High**	23%	52%	28%	6%
**Occupation**				
** Unskilled**	44%	16%	40%	62%
** Homemaker**	9%	3%	2%	16%
** Skilled/clerical**	23%	33%	29%	13%
** Manager/professional**	24%	48%	28%	9%
**Reproductive history factors**				
** Age menarche**	13.3 (2.9)	13.1 (2.1)	13.0 (2.2)	13.6 (2.5)
** Age menopause**	47.1 (7.9)	48.6 (6.9)	45.6 (8.6)	47.3 (7.9)
** Reproductive span**	33.8 (8.3)	35.5 (7.4)	32.6 (8.5)	33.8 (8.3)
** Number of births**	2.5 (2.8)	1.5 (1.7)	1.8 (2.0)	3.6 (3.2)
** Ever breastfed, % yes**	81%	69%	75%	90%
** Years breastfed**	1.6 (3.3)	0.60 (1.6)	0.78 (1.7)	2.6 (4.2)
** Ever HRT, % yes**	16%	29%	13%	13%
** Years HRT**	0.92 (3.8)	2.3 (6.5)	0.66 (3.3)	0.44 (2.2)
** Estrogen exposure index**	33.2 (9.9)	37.2 (10.1)	32.5 (9.1)	31.6 (9.2)

*Note.* HRT = hormone replacement therapy. Mean (standard deviation) or % are reported.

### Model 1: Associations of the estrogen exposure index and individual reproductive history indicators with cognitive outcomes

Overall, associations were consistent with expectations. Greater lifetime exposure to estrogen was associated with higher baseline memory performance (*b* = 0.023, 95% CI: 0.016, 0.030), slower memory decline (*b* = 0.014, 95% CI: 0.003, 0.026), and a lower risk of dementia (HR = 0.947, 95% CI: 0.924, 0.969). Longer reproductive span and duration of HRT use were associated with higher baseline memory performance (reproductive span: *b* = 0.012, 95% CI: 0.003, 0.020; HRT use: *b* = 0.010, 95% CI: 0.005, 0.014), and longer duration of HRT use was also associated with a slower rate of memory decline (*b* = 0.009, 95% CI: 0.004, 0.015). Later age at menarche and longer duration of breastfeeding were both associated with lower baseline memory performance (menarche age: *b* = −0.038, 95% CI: −0.056, −0.021; breastfeeding duration: *b* = −0.010, 95% CI: −0.013, −0.008) and a higher risk of dementia (menarche age: HR = 1.14, 95% CI: 1.06, 1.22; breastfeeding duration: HR = 1.02, 95% CI: 1.01, 1.03).

Across racial and ethnic groups (Table 2), greater lifetime exposure to estrogen was associated with higher baseline memory performance among Black and Latinx women, slower memory decline among White women, and lower dementia risk among Latinx women. Among Black women, an older age at menarche was associated with lower baseline memory performance, faster memory decline, and higher dementia risk. Older age at menarche was also associated with lower baseline memory performance and increased dementia risk among Latinx women. Latinx and Black women with longer reproductive spans demonstrated higher baseline memory performance. Longer duration of breastfeeding was associated with lower baseline memory performance among Latinx women and increased dementia risk among Latinx and White women. Longer duration of HRT use was reliably associated with higher baseline memory performance among Black women and slower memory decline among White women. Estimates of associations between reproductive history factors and each outcome were not reliably different across racial and ethnic groups, using a *p*-value threshold of .01 (99% CI) or the less conservative *p*-value threshold of .05 (95% CI).

**Table 2.** Associations of estrogen exposure variables with baseline memory performance, rate of memory decline, and dementia risk across racial and ethnic groups.

Variable	Model 1		
	White women	Black women	Latinx women
	b or HR (95% CI)	b or HR (95% CI)	b or HR (95% CI)
**Baseline memory**			
** Estrogen exposure index**	–0.004 (–0.015, 0.007)	0.013 (0.002, 0.023)	0.014 (0.004, 0.024)
** Age at menarche**	–0.008 (–0.039, 0.023)	–0.038 (–0.054, –0.022)	–0.018 (–0.034, –0.002)
** Age at menopause**	–0.003 (–0.013, 0.007)	0.002 (–0.004, 0.008)	0.003 (–0.003, 0.009)
** Reproductive span**	0.012 (0.003, 0.020)	0.007 (0.000, 0.014)	0.008 (0.001, 0.015)
** Duration HRT use**	0.001 (–0.003, 0.004)	0.009 (0.003, 0.015)	0.004 (–0.008, 0.016)
** Duration breastfed**	0.001 (–0.013, 0.016)	–0.002 (–0.015, 0.011)	–0.005 (–0.008, –0.002)
**Memory decline**			
** Estrogen exposure index**	0.021 (0.005, 0.037)	0.002 (–0.016, 0.019)	0.009 (–0.003, 0.021)
** Age at menarche**	0.004 (–0.042, 0.051)	–0.042 (–0.068, –0.016)	–0.005 (–0.031, 0.021)
** Age at menopause**	0.006 (–0.009, 0.022)	–0.003 (–0.015, 0.009)	0.004 (–0.006, 0.013)
** Reproductive span**	0.012 (–0.011, 0.035)	–0.002 (–0.020, 0.016)	0.010 (–0.009, 0.029)
** Duration HRT use**	0.007 (0.002, 0.012)	0.005 (–0.004, 0.014)	0.003 (–0.004, 0.010)
** Duration breastfed**	–0.008 (–0.017, 0.001)	0.004 (–0.011, 0.019)	0.001 (–0.005, 0.007)
**Dementia risk**			
** Estrogen exposure index**	0.965 (0.916, 1.01)	0.981 (0.933, 1.03)	0.961 (0.930, 0.992)
** Age at menarche**	1.11 (0.878, 1.33)	1.18 (1.05, 1.31)	1.07 (1.01, 1.13)
** Age at menopause**	1.01 (0.933, 1.08)	0.999 (0.971, 1.03)	0.992 (0.974, 1.01)
** Reproductive span**	0.998 (0.898, 1.10)	0.983 (0.942, 1.02)	0.975 (0.950, 1.00)
** Duration HRT use**	0.857 (0.614, 1.10)	0.989 (0.935, 1.04)	0.962 (0.861, 1.06)
** Duration breastfed**	1.03 (1.00, 1.06)	1.02 (.901, 1.14)	1.05 (1.01, 1.09)

*Note.* HR = hazard ratios; HRT = hormone replacement therapy. Model 1 = examined independent associations between the estrogen exposure variables and cognitive outcomes, and accounts for age at baseline, age squared, APOE-ε4 status, and body mass index at baseline. B is reported for baseline memory and memory decline; HR is reported for dementia risk.

### Model 2: Associations between lifecourse social factors and estrogen exposure indicators

Associations between lifecourse social exposures and the EEI, as well as each reproductive history factor, are presented in Supplementary Table 2. In general, indicators of higher SES were associated with a higher EEI, younger age at menarche, longer reproductive span, shorter duration of breastfeeding, and longer duration of HRT use. A later birth year was also associated with a higher EEI, a younger age at menarche, and a longer reproductive span, as well as an older age at menopause and a shorter duration of breastfeeding.

**Figure 1.**
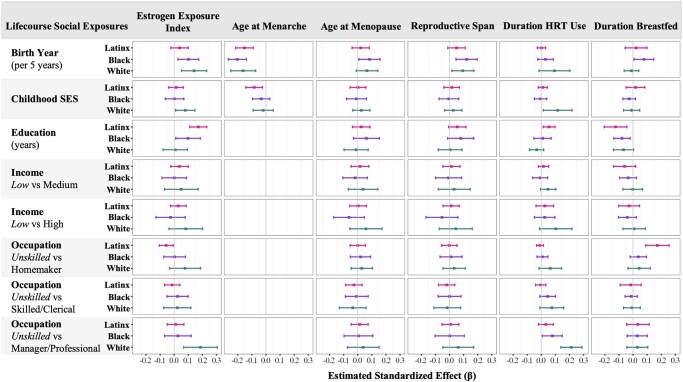
Associations of lifecourse social exposures and estrogen exposure variables across racial and ethnic groups. The figure plots standardized effect estimates (*x*-axis) of associations between each lifecourse social exposure (rows) and each estrogen exposure variable (columns). HRT = Hormone Replacement Therapy; SES = Socioeconomic Status.

Lifecourse social experiences were differentially associated with the EEI across racial and ethnic groups (Figure 1). Every 5-year increase in birth year was reliably associated with greater lifetime estrogen exposure among White and Black women. Higher educational attainment was associated with greater estrogen exposure among Black women and Latinx women. This association was stronger for Latinx women compared with White women (difference in unstandardized beta [*b*_diff_] = −0.151, 99% CI: −0.272, −0.029). Compared to unskilled occupations, White women who held managerial or professional occupations had higher levels of lifetime estrogen exposure, and Latinx women who were homemakers most of their lives had lower estrogen exposure levels. The association between managerial/professional occupation and cumulative estrogen exposure was stronger among White women compared with Latinx women (*b*_diff_ = 1.45, 99% CI: 0.134, 2.76).

Examination of relationships between lifecourse social exposures and individual reproductive history factors revealed that later birth year was associated with earlier age at menarche, later age at menopause, and longer reproductive span across racial and ethnic groups (Figure 1). Later birth year was also associated with longer duration of HRT use among White women and longer duration of breastfeeding among Black women. Higher childhood SES was associated with earlier age at menarche among Latinx women and longer duration of HRT use among White women. Women with higher educational attainment reported fewer years of breastfeeding. Reproductive span was longer among Black and Latinx women with higher educational attainment. Among Latinx women, higher educational attainment was associated with longer duration of HRT use. Compared to White women with low monthly income, White women with high income had a longer duration of HRT. Compared to unskilled occupations, White and Black women who held managerial or professional occupations reported longer HRT use, and Latinx women who were homemakers most of their lives had a longer duration of breastfeeding.

### Model 3: Joint influence of lifecourse social factors and estrogen exposure indicators on cognitive outcomes

For parsimony in Model 3, we combined the childhood and adult SES indicators into a latent factor representing lifecourse SES. Supplementary Figure 3 illustrates the hypothesized pathways linking the lifecourse social factors—birth year and lifecourse SES—to cognitive outcomes via the EEI. Supplementary Table 3 presents model estimates for each outcome in the entire sample. Path coefficients for associations between birth year, lifecourse SES, and the EEI were nearly identical across the latent growth curve and Cox regression models. We found that birth year was indirectly associated with the EEI (*b* = 0.261, 95% CI: 0.211, 0.311), baseline memory performance (*b* = 0.063, 95% CI: 0.054, 0.072), memory decline (*b* = 0.018, 95% CI: 0.009, 0.027), and dementia risk (HR = 0.689, 95% CI: 0.637, 0.745) via lifecourse SES; therefore, mediation analyses focused on the lifecourse SES factor as the main exposure. A detailed decomposition of mediation effects for each mediator, outcome, and racial and ethnic group is presented in Supplementary Tables 3–5.

**Figure 2.**
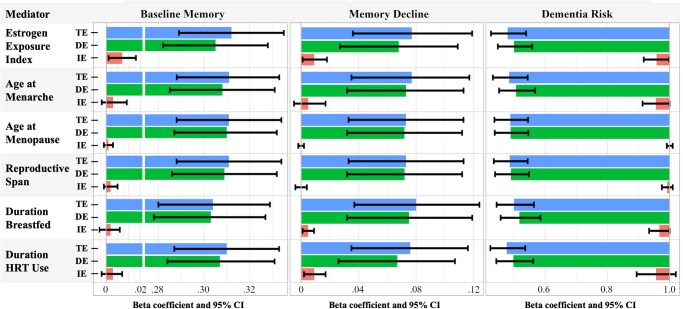
Estimated total effects, natural direct effects, and total natural indirect effects from mediation analyses in the entire sample. The figure shows beta coefficients and hazard ratios for mediation analyses. DE = the Pure Natural Direct Effect of lifecourse SES that is not mediated; HRT = Hormone Replacement Therapy; SES = Socioeconomic Status; IE = the Total Natural Indirect Effect of lifecourse SES via the mediator; TE = Total Effect of lifecourse SES on an outcome.

Figure 2 shows TE, direct effect, and indirect effect estimates for each mediation model. The proportion of the TE of lifecourse SES mediated by the EEI was 2.2% for baseline memory, 11.6% for memory decline, and 6% for dementia risk. Decomposition of these indirect effects (Supplementary Table 3) shows that the PNIE is statistically significant for each outcome. Mediated interactions (INTmed) are also present, suggesting that the overall mediated effect is due to the combination of lifecourse SES influencing estrogen exposure and the association of estrogen exposure on the outcome being stronger for women with higher lifecourse SES compared to those with lower lifecourse SES.

The decomposition of the indirect effect of lifecourse SES on memory decline through duration of HRT use (Figure 2; Supplementary Table 4) was similar to that of the EEI, where 11.8% of the TE of lifecourse SES on memory decline is due to the combination of lifecourse SES influencing HRT use and the association of HRT use with memory decline being stronger among women with higher lifecourse SES. In models that included menarche age as a mediator (Figure 2; Supplementary Table 4), the PNIE for dementia risk is small but statistically significant, suggesting that a small portion (6%) of the positive association between higher lifecourse SES and dementia risk may operate through menarcheal timing. The overall mediation effect of lifecourse SES on memory decline via breastfeeding duration (accounting for the number of births) was statistically significant (Figure 2); however, this effect appears to be entirely driven by the mediated interaction (Supplementary Table 4). Indirect effects in models with menopause age or reproductive span as the mediator accounted for less than 1% of the TE of lifecourse SES on respective outcomes.

**Figure 3.**
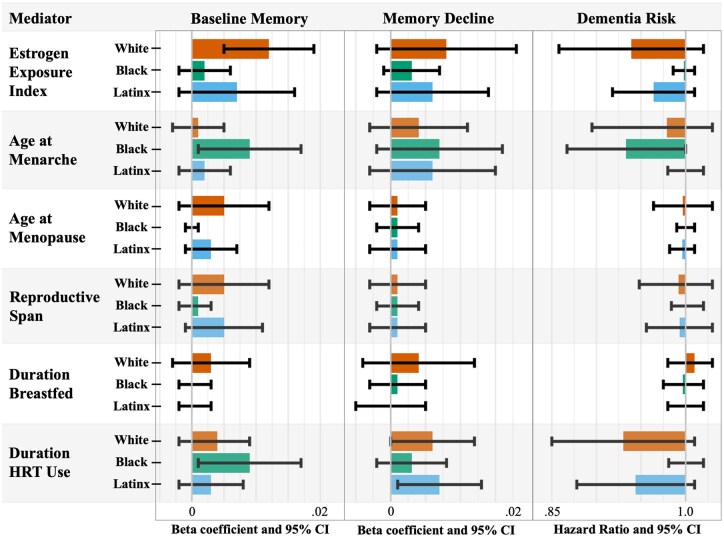
Estimated natural indirect effects from mediation analyses across racial and ethnic groups. The figure shows beta coefficients and hazard ratios from mediation analyses. Only indirect effects are presented. HRT = Hormone Replacement Therapy; SES = Socioeconomic Status.

Estimated indirect effects across racial and ethnic groups (Figure 3) revealed that among White women, the EEI mediated a significant portion of the lifecourse SES-baseline memory performance relationship (4.4% mediated). HRT use mediated associations of lifecourse SES with baseline memory among Black women (3.4%) and memory decline among White (4.3%) and Latinx women (8.2%). Age at menarche was a significant mediator for baseline memory and dementia risk for Black women (3.6%–8%).

Finally, we reestimated Model 3 with birth year as a moderator rather than an exposure variable. Multiple group analyses for two birth year groups (birth before 1926 vs. birth after) found similar estimates for Model 3 across the two cohorts. Because place of birth cannot be examined across racial and ethnic groups in the WHICAP sample, we determined whether place of birth influenced results in models stratified by race and ethnicity. Among Latinx women, birth in Puerto Rico or Cuba versus the DR was not associated with any of the estrogen exposure indicators or cognitive outcomes. Among Black women, birth in a southern state was indirectly associated with baseline memory performance via lifecourse SES and was not significantly related to the EEI. Similarly, among White women, birth in a European country versus the United States was not associated with the EEI but was indirectly associated with baseline memory via lifecourse SES. Inclusion of these birthplace indicators did not influence the results reported above. Furthermore, we found no interactions between APOE e4 status and reproductive history factors across racial and ethnic groups.

## Discussion

In this community-based sample of postmenopausal women, we investigated the complex relationships between cumulative estrogen exposure, life course social exposures, and late-life cognitive health. We found that later birth year and higher lifecourse SES were associated with greater cumulative estrogen exposure in later life. Estrogen exposure indicators were independently associated with memory trajectories and dementia risk across racial and ethnic groups. Associations between lifecourse SES and each cognitive outcome were partially mediated by estrogen exposure indicators.

Findings from this study provide further evidence that lifetime cumulative estrogen exposure is socially patterned. Consistent with previous work (Nabhan et al., 2022), a later birth year was associated with an earlier age at menarche across racial and ethnic groups. We also found that higher childhood SES was associated with earlier menarche among Latinx women. Although data from the Caribbean are lacking, studies in other Latin American countries found that higher childhood SES is associated with a younger age at menarche and attributed the decline in age at menarche over time to improved social and living conditions for young girls (Leone & Brown, 2020). Net of occupational attainment and income, we found positive associations between educational attainment and lifetime cumulative estrogen exposure among Black and Latinx women, driven by associations of education with reproductive span and HRT use. Education may be linked to these reproductive health factors via increased health literacy. For example, knowledge of menopausal symptoms can influence the timing of seeking medical care and initiating HRT (Suka et al., 2016). Trust in healthcare providers and the skills needed to navigate healthcare systems and institutions may also influence decision-making to undergo medical treatments that cause early menopause, such as oophorectomy, hysterectomy, and some cancer treatments (Brennand et al., 2024). We also found that higher educational attainment was associated with a shorter duration of breastfeeding across racial and ethnic groups. Most recent U.S.-based studies show that higher educational attainment is positively associated with breastfeeding duration (Dagher et al., 2016). However, many women in this study gave birth sometime during the 1940s to 1980s. During this period, the United States saw a decline in breastfeeding rates and increased use of infant formula among women with higher educational attainment (Institute of Medicine (US) Committee on Nutritional Status During Pregnancy and Lactation, 1991), and this trend may explain our findings.

Our findings suggest that cumulative estrogen exposure acts as a significant biological mechanism linking a woman’s social experiences to her cognitive health. The EEI mediated small but significant proportions of the TE of lifecourse SES on baseline memory, memory decline, and dementia risk. The overall mediation effect (TNIE) captures both the simple mediated pathway and the interaction between the exposure and mediator. The presence of a mediated interaction effect (INTmed > 0) suggests that lifecourse SES not only has an indirect impact on cognitive outcomes through estrogen exposure but also interacts with estrogen exposure in a nuanced way. One potential reason for this is that higher lifecourse SES may shape access to cognitive reserve-enhancing lifecourse experiences (e.g., educational attainment, occupational complexity, etc.) that buffer the negative impact of brain pathology on cognitive decline (Stern, 2002). This cognitive reserve may, in turn, enhance the neuroprotective effects of estrogen exposure in later life.

Women with higher lifecourse SES were more likely to use HRT and for a longer duration than their counterparts with lower lifecourse SES. In turn, longer HRT use was positively associated with cognitive outcomes. Despite racial and ethnic differences in overall use, HRT mediated lifecourse SES-cognitive outcome relationships for Black, White, and Latinx women in the study. Regardless of SES, White women are more likely than their Black and Latinx counterparts to be prescribed HRT (Khalil et al., 2024). Although the current study’s findings provide support for a potential beneficial influence of HRT use, the evidence for late-life cognitive benefits associated with HRT is mixed. Observational studies linked HRT use to a reduced dementia risk (Stute et al., 2021); however, clinical trials have either shown no cognitive benefits from HRT (Gleason et al., 2015) or harmful effects, including increased dementia risk (Shumaker et al., 2004). These differences may be due to observational study findings being the result of confounding (i.e., HRT users are more likely to have higher SES and are therefore at lower risk for cognitive decline or dementia) (Mielke et al., 2014). Our findings indicate that lifecourse SES not only affects HRT use but also alters how HRT affects cognition, as we observed that the positive effect of HRT on cognitive decline is greater for women with higher lifecourse SES compared to those with lower SES. HRT type, dosages, and age at initiation may also influence associations with cognitive health outcomes (Stute et al., 2021). Information on these factors was not consistently collected from HRT users in the current study, limiting the scope of our findings.

Among Black women in this study, associations of higher lifecourse SES with baseline memory and dementia risk were partially mediated through menarcheal timing. Like most older Black adults in the United States (Ruggles et al., 2004), a majority of the Black women in WHICAP were born and raised in the South, where Jim Crow laws limited health-enhancing opportunities and resources for young Black girls and their families (Thomas, 2011). Future research will benefit from including measures of these macrolevel structural determinants when examining the link between age at menarche and late-life cognitive health among Black women. Moreover, most of the research linking menarche age to adult health outcomes among Black women has focused on breast cancer risk and found later age at menarche to be protective (Ambrosone et al., 2015). More research is needed in this area to understand if the timing of menarche has a differential impact on midlife versus later-life diseases, independent of other reproductive history factors (i.e., age and type of menopause, reproductive span).

The current study did not differentiate results across reported types of menopause (i.e., natural versus surgical) or history of reproductive surgery (e.g., hysterectomy with ovarian sparing) because only 20% of the sample provided this information. Each of these factors may be associated with distinct hormonal change trajectories before and after menopause (Edwards et al., 2019) that should be considered when estimating lifetime cumulative estrogen exposure. There is also a long history of forced sterilization of Black and Latinx women in the United States via hysterectomy (Downham Moore, 2023) that likely contributes to racial and ethnic differences in cumulative estrogen exposure. Future research will benefit from examining associations between lifecourse social exposures, cumulative estrogen exposure, and cognitive health outcomes across different reproductive histories.

Our measure of monthly income was based on self-report, and classification of each income category was arbitrarily decided at the beginning of the study in 1992. The cognitive health benefits associated with a particular income category in 1992 may not be associated with that same income category for the 1999 and 2009 cohorts. Also, income was measured at participants’ baseline study visit and likely did not represent their income before having children or the menopausal transition. Alternatives to monthly income should be included in future research that accounts for inflation, household size, and wealth across the lifecourse (Williams et al., 2010). Key lifecourse social exposures related to memory trajectories and dementia risk were not captured with the measures used in this study, which may also be associated with lifetime cumulative estrogen exposure, including structural-level discrimination, residential history, access to medical care, and health literacy. An individual’s lifecourse trajectory, the opportunities and constraints they experience are shaped by historical time and place (Haas et al., 2017). Birthplace and time are contextual indicators of a range of lifecourse exposures that may shape both reproductive history and cognitive health outcomes. In the current study, however, these indicators were mediated entirely by lifecourse SES. To develop a more precise understanding of how social exposures influence reproductive health across the lifecourse, future studies must leverage data from larger representative studies with multiple age cohorts.

A major strength of this study is the use of multiple imputation to account for missing reproductive history and lifecourse social exposure data. Most research linking reproductive health to late-life cognition excludes participants with missing reproductive history data. Such a practice reduces sample size and can bias observed associations by distorting the representation of the sample population (Kang, 2013). In the current study, data were assumed to be MAR (i.e., the probability of missingness in the current study was influenced by observed variables such as age and study cohort), which enabled us to use multiple imputation analyses to produce unbiased results with proper confidence intervals and maintain power that otherwise would have been lost.

Although it is increasingly recognized that research to improve women’s cognitive health outcomes requires considering both biological and social influences (National Academies of Sciences, Engineering, and Medicine; Health and Medicine Division; Board on Population Health and Public Health Practice; Committee on Assessment of NIH Research on Women’s Health, 2024), research in this area has continued to treat sex-linked biology and social influences as separate entities. This study provides compelling support that cumulative estrogen exposure and lifecourse social exposures jointly influence women’s late-life memory trajectories and dementia risk. Our results suggest that the pathways linking lifecourse social factors to cognitive health outcomes via estrogen exposure are complex and due to a combination of interactions and indirect effects. A deeper understanding of how sex-linked biological mechanisms and gender-related social forces intertwine is essential for the development of interventions that will decrease AD risk among women.

## Supplementary Material

gbag001_Supplementary_Data

## Data Availability

The de-identified individual participant data used in this study is not preregistered but can be accessed by submitting a paper proposal for the Washington Heights-Inwood Columbia Aging Project (WHICAP).
