# Supplementary material for: Associations of lifetime cumulative estrogen exposure with lifecourse social exposures, cognitive decline, and dementia risk among postmenopausal White, Black, and Latina women
Source: J Gerontol B Psychol Sci Soc Sci. 2026 Jan 10;81(3):gbag001. doi: 10.1093/geronb/gbag001 (PMC12997449; doi:10.1093/geronb/gbag001)
Supplement: gbag001_Supplementary_Data [file gbag001_supplementary_data.docx]

***The Journals of Gerontology, Series B: Psychological Sciences and Social Sciences* Supplementary Material: Avila-Rieger et al. Associations of Lifetime Cumulative Estrogen Exposure with Lifecourse Social Exposures, Cognitive Decline, and Dementia Risk Among Postmenopausal White, Black, and Latina Women.**

**Supplementary Methods**

**Missingness and Multiple Imputation**

Of the 3,688 women included in the study, 1,205 were missing at least one of the reproductive health variables used to construct the lifetime cumulative estrogen exposure index, and 291 were missing at least one lifecourse social exposure variable (**Supplementary Table 1**).

We used the R package Multivariate Imputation by Chained Equations (MICE) for multiple imputation. Missing continuous variables were imputed via predictive mean matching, missing binary variables via logistic regression, and missing unordered categorical variables via polytomous regression. Each missing value was imputed with simulated possible values. We generated 10 imputed data sets with an imputation model that included all lifecourse social exposures, reproductive health variables, cognitive outcomes, and covariates to be analyzed. Estimates were pooled for each model. Convergence and diagnostics are presented in **Supplementary Figures 1 and 2**.

**Supplementary Table 1.** Participant Characteristics: Non-Imputed Data

|  | **Full**  **Sample** | **White**  **Women** | **Black**  **Women** | **Latinx**  **Women** |
| --- | --- | --- | --- | --- |
| **N** | 3688 | 840 | 1177 | 1671 |
| **Age at Baseline^a,b^** | 75.9 (6.6) | 76.4 (7.1) | 76.1 (6.6) | 75.6 (6.2) |
| **Birth Year^a,b^** | 1927 (11) | 1926 (11) | 1927 (11) | 1928 (10) |
| **Birthplace, % U.S. Born^b^** | 45 | 60 | 92 | 3 |
| **APOE-ε4 Status, % positive** | 27 | 22 | 34 | 25 |
| *% Missing* | *11* | *11* | *14* | *10* |
| **Body Mass Index (BMI)^a^** | 28.8 (6.1) | 26.7 (5.6) | 29.6 (7.0) | 29.3 (5.5) |
| *% Missing* | *14* | *13* | *16* | *12* |
| **Childhood SES^a^** | 0.01 (0.9) | 0.49 (0.8) | 0.11 (0.8) | -0.28 (0.8) |
| *% Missing* | *7* | *9* | *8* | *6* |
| **Educational Attainment^a^** | 10.1 (4.9) | 13.7 (3.5) | 11.8 (3.6) | 6.9 (4.4) |
| *% Missing* | *0.7* | *0.6* | *0.9* | *0.5* |
| **Income, %** |  |  |  |  |
| Low | 42 | 16 | 32 | 60 |
| Medium | 35 | 31 | 40 | 34 |
| High | 23 | 53 | 28 | 6 |
| *% Missing* | *8* | *16* | *8* | *4* |
| **Occupation, %** |  |  |  |  |
| Unskilled | 45 | 16 | 40 | 62 |
| Homemaker | 9 | 3 | 2 | 16 |
| Skilled/Clerical | 23 | 33 | 29 | 13 |
| Manager/ Professional | 24 | 48 | 28 | 9 |
| *% Missing* | *4* | *3* | *3* | *4* |
| **Reproductive History Factors** |  |  |  |  |
| Age Menarche^a^ | 13.2 (1.8) | 12.9 (1.5) | 12.9 (1.7) | 13.5 (1.9) |
| *% Missing* | *21* | *21* | *23* | *19* |
| Age Menopause^a^ | 47.1 (7.3) | 48.6 (6.0) | 45.6 (8.0) | 47.3 (7.1) |
| *% Missing* | *12* | *13* | *13* | *12* |
| Reproductive Span^a^ | 33.9 (7.4) | 35.8 (6.1) | 32.7 (8.3) | 33.8 (7.2) |
| *% Missing* | *25* | *25* | *27* | *23* |
| Number of Births^a^ | 2.5 (2.5) | 1.4 (1.5) | 1.8 (1.9) | 3.5 (2.9) |
| *% Missing* | *18* | *16* | *20* | *18* |
| Years Breastfed^a^ | 1.2 (2.4) | 0.42 (0.8) | 0.61 (1.1) | 2.5 (3.5) |
| *% Missing* | *49* | *35* | *47* | *59* |
| Years HRT^a^ | 0.93 (3.7) | 2.3 (5.8) | 0.68 (3.1) | 0.43 (2.1) |
| *% Missing* | *15* | *14* | *14* | *16* |
| Estrogen Exposure Index^a^ | 33.0 (8.7) | 37.1 (8.6) | 31.9 (7.8) | 30.7 (8.4) |
| *% Missing* | *57* | *45* | *55* | *65* |

^a^ Data are shown as mean (SD); ^b^ no missing data

**Supplementary Figure 1.** Means and standard deviations of synthetic values plotted against iteration number for imputed data


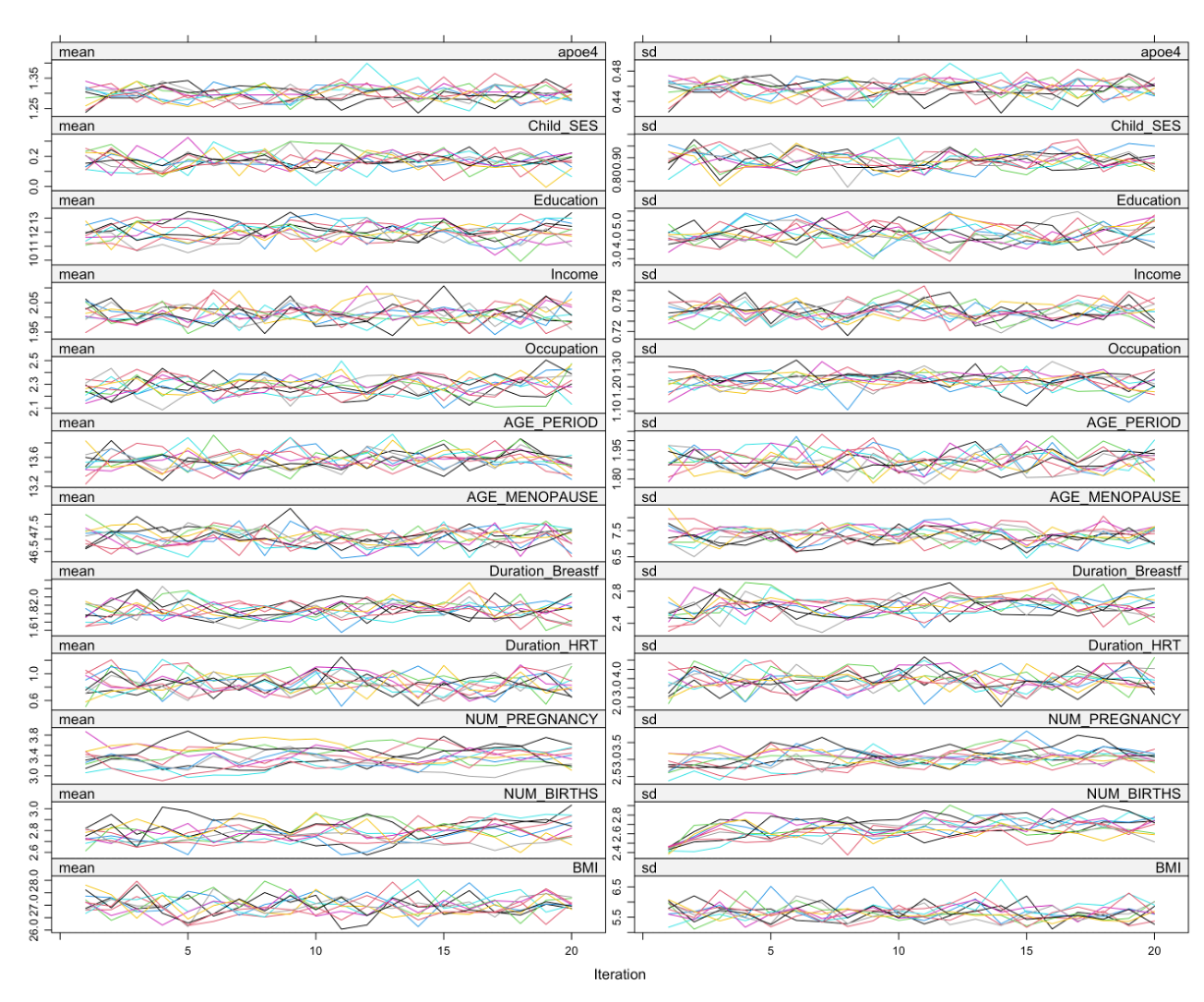


**Supplementary Figure 2.** Kernel density estimates for the marginal distributions of observed data (blue line) and densities per variable calculated (red lines)


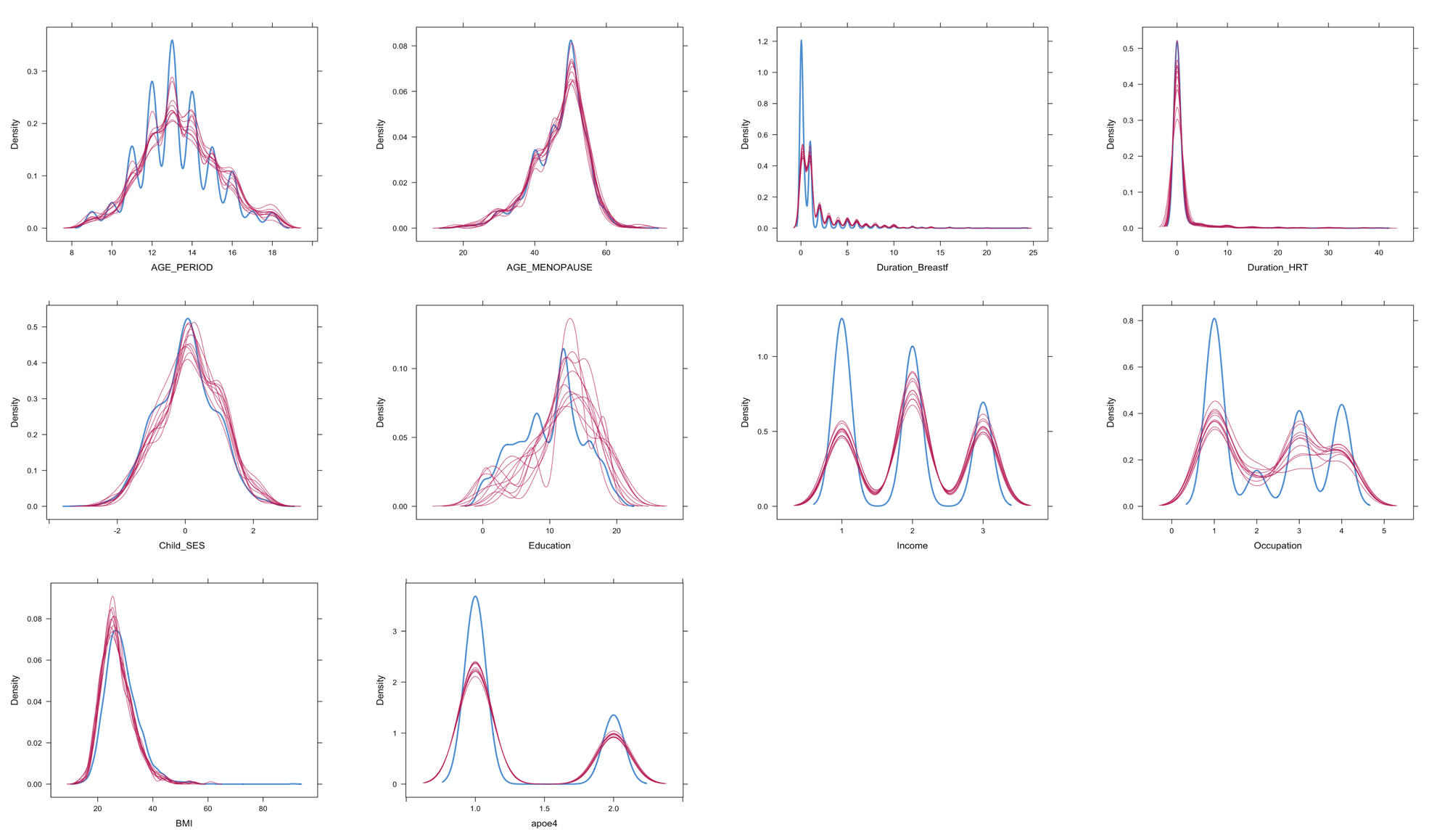


**Supplementary Table 2.** Associations of Lifecourse Social Exposures and Estrogen Exposure Variables Across Entire Sample

| **Lifecourse Social Exposure** | **Cumulative Estrogen Exposure** | **Age at Menarche** | **Age at Menopause** | **Reproductive Span** | **Duration HRT Use** | **Duration Breastfed** |
| --- | --- | --- | --- | --- | --- | --- |
|  | β (95% CI) | β (95% CI) | β (95% CI) | β (95% CI) | β (95% CI) | β (95% CI) |
| Birth Year | .063 (.013, .113) | -.160 (-.223, -.097) | .057 (.007, .107) | .085 (.034, .136) | .035 (-.017, .087) | .083 (.021, .146) |
| Childhood SES | .053 (.003, .104) | -.099 (-.148, -.050) | .018 (-.033, .069) | .027 (-.024, .078) | .059 (.012, .107) | -.016 (-.066, .033) |
| Education | .165 (.102, .227) |  | .012 (-.053, .076) | .049 (-.015, .113) | .049 (-.001, .100) | -.139 (-.213, -.064) |
| Income (ref. Low) |  |  |  |  |  |  |
| Medium | .034 (-.022, .091) |  | .005 (-.054, .063) | .008 (-.052, .069) | .014 (-.029, .057) | -.071 (-.146, .004) |
| High | .064 (-.001, .128) |  | .010 (-.061, .081) | .014 (-.057, .084) | .078 (-.004, .160) | -.062 (-.127, .002) |
| Occupation (ref. Unskilled) |  |  |  |  |  |  |
| Homemaker | -.015 (-.061, .031) |  | .024 (-.022, .070) | .020 (-.027, .068) | .023 (-.018, .064) | .141 (.071, .212) |
| Skilled/Clerical | .002 (-.051, .056) |  | -.020 (-.077, .037) | -.010 (-.066, .047) | .011 (-.035, .057) | -.021 (-.073, .032) |
| Manager/Professional | .065 (.002, .129) |  | .031 (-.040, .101) | .032 (-.036, .099) | .125 (.056, .194) | .051 (-.020, .122) |

β = Estimated standardized effect.

**Supplementary Figure 3.** Hypothesized pathways linking the lifecourse social factors – birth year and lifecourse SES - to cognitive outcomes via the estrogen exposure index.

**Supplementary Table 3.** Estimates from Model 3 across the entire sample.

|  |  |  | **Model 3** | | | |
| --- | --- | --- | --- | --- | --- | --- |
|  |  |  | **Latent Growth Curve Model** | |  | **Cox Regression Model** |
| **Direct Effects** | **Path** |  | b (95% CI) | |  | b (95% CI) |
| BY 🡪 EEI | a1 |  | .213 (.188, .238) | |  | .213 (.188, .238) |
| BY 🡪 LSES | a2 |  | .221 (.061, .380) | |  | .220 (.060, .379) |
| LSES 🡪 EEI | a3 |  | .859 (.696, 1.03) | |  | .859 (.696, 1.04) |
|  |  |  | **Baseline memory** | **Memory decline** |  | **Dementia Risk** |
|  |  |  | b (95% CI) | b (95% CI) |  | HR (95% CI) |
| EEI 🡪 Y | b1 |  | .006 (.000, .011) | .008 (-.001, .017) |  | .983 (.966, 1.00) |
| BY 🡪 Y | c1 |  | .039 (.012, .065) | .031 (-.023, .084) |  | 1.07 (.921, 1.22) |
| LSES 🡪 Y | c2 |  | .305 (.282, .329) | .068 (.027, .109) |  | .500 (.448, .559) |
| **Decomposition** |  |  |  |  |  |  |
| LSES 🡪 EEI 🡪 Y | |  |  |  |  |  |
| TE |  |  | .312 (.289, .335) | .077 (.036, .119) |  | .479 (.426, .539) |
| PNDE |  |  | .305 (.282, .328) | .068 (.027, .109) |  | .500 (.448, .559) |
| TNIE |  |  | .007 (.001, .013) | .009 (.001, .018) |  | .958 (.917, .999) |
| PNIE |  |  | .004 (.000, .009) | .006 (.000, .014) |  | .969 (.945, .994) |
| INTmed |  |  | .003 (-.002, .007) | .003 (-.004, .011) |  | .989 (.978, 1.00) |

BY = Birth Year; LSES = Lifecourse SES; EEI = Estrogen Exposure Index; TE = Total Effect; PNDE = the portion of the TE that is not mediated through the EEI; TNIE = portion of the TE transmitted through the EEI; PNIE = the indirect effect solely due to the mediated pathway; INTmed = the portion of the TNIE that results from the exposure-mediator interaction.

**Supplementary Table 4.** Decomposition of mediation effects for each estrogen exposure indicator across the entire sample.

|  | **Baseline Memory** | **Memory Decline** | **Dementia Risk** |
| --- | --- | --- | --- |
|  | b (95% CI) | b (95% CI) | HR (95% CI) |
| **LSES 🡪 Menarche Age 🡪 Y** |  |  |  |
| TE | .311 (.288, .334) | .077 (.035, .117) | .484 (.432, .544) |
| PNDE | .308 (.285, .331) | .073 (.032, .113) | .507 (.452, .568) |
| TNIE | .003 (-.002, .009) | .005 (-.005, .017) | .956 (.913, 1.00) |
| PNIE | .002 (-.002, .006) | .003 (-.004, .010) | .969 (.944, .993) |
| INTmed | .001 (-.002, .005) | .002 (-.005, .008) | .987 (.874, 1.10) |
| **LSES 🡪 Menopause Age 🡪 Y** |  |  |  |
| TE | .311 (.288, .333) | .073 (.033, .113) | .488 (.437, .545) |
| PNDE | .310 (.287, .332) | .072 (.032, .112) | .489 (.438, .546) |
| TNIE | .001 (-.001, .003) | .000 (-.002, .002) | .998 (.991, 1.01) |
| PNIE | .000 (.000, .001) | .000 (-.001, .003) | .998 (.994, 1.01) |
| INTmed | .001 (.000, .002) | .001 (.000, .003) | .999 (.982, 1.01) |
| **LSES 🡪 Reproductive Span 🡪 Y** |  |  |  |
| TE | .311 (.288, .334) | .073 (.033, .113) | .486 (.435, .544) |
| PNDE | .309 (.286, .332) | .072 (.032, .112) | .490 (.439, .548) |
| TNIE | .002 (-.001, .005) | .000 (-.004, .004) | .992 (.975, 1.01) |
| PNIE | .001 (-.001, .003) | .002 (-.001, .005) | .993 (.984, 1.01) |
| INTmed | .001 (-.001, .003) | -.002 (-.005, .001) | .999 (.982, 1.01) |
| **LSES 🡪 Breastfed 🡪 Y** |  |  |  |
| TE | .304 (.280, .329) | .080 (.037, .124) | .500 (.444, .564) |
| PNDE | .303 (.278, .327) | .075 (.032, .119) | .517 (.457, .585) |
| TNIE | .002 (-.003, .006) | .005 (.001, .009) | .967 (.934, 1.00) |
| PNIE | .001 (-.001, .004) | .001 (-.004, .007) | .980 (.960, 1.00) |
| INTmed | .000 (-.002, .003) | .004 (.000, .010) | .985 (.970, 1.00) |
| **LSES 🡪 HRT Use 🡪 Y** |  |  |  |
| TE | .310 (.287, .333) | .076 (.035, .116) | .477 (.424, .536) |
| PNDE | .307 (.284, 331) | .067 (.026, .107) | .498 (.443, .562) |
| TNIE | .003 (-.002, .007) | .009 (.002, .017) | .957 (.894, 1.02) |
| PNIE | .003 (-.003, .009) | .006 (-.002, .015) | .979 (.945, 1.01) |
| INTmed | .000 (-.005, .005) | .003 (-.006, .011) | .977 (.934, 1.03) |

LSES = Lifecourse SES; TE = Total Effect; PNDE = the portion of the TE that is not mediated through the EEI; TNIE = portion of the TE transmitted through the EEI; PNIE = the indirect effect solely due to the mediated pathway; INTmed = the portion of the TNIE that results from the exposure-mediator interaction.

**Supplementary Table 5.** Decomposition of mediation effects for each estrogen exposure indicator across racial and ethnic groups

|  | **Baseline memory** | **Memory decline** | **Dementia Risk** |
| --- | --- | --- | --- |
| **White Women** | b (95% CI) | b (95% CI) | HR (95% CI) |
| **LSES 🡪 EEI 🡪 Y** |  |  |  |
| TE | .272 (.201, .344) | .134 (.005, .262) | .476 (.307, .738) |
| PNDE | .253 (.181, .327) | .125 (-.005, .253) | .507 (.330, .779) |
| TNIE | .012 (.005, .019) | .008 (-.003, .026) | .939 (.852, 1.03) |
| PNIE | .006 (-.001, .018) | .007 (-.013, .033) | .990 (.916, 1.07) |
| INTmed | .006 (-.006, .021) | .001 (-.022, .026) | .948 (.886, 1.01) |
| **LSES 🡪 Menarche Age 🡪 Y** |  |  |  |
| TE | .266 (.195, .338) | .143 (.016, .273) | .502(.324, .781) |
| PNDE | .265 (.193, .336) | .138 (.010, .267) | .513 (.333, .792) |
| TNIE | .001 (-.003, .005) | .004 (-.006, .021) | .979 (.895, 1.07) |
| PNIE | .000 (-.008, .008) | .003 (-.011, .026) | 1.00 (.938, 1.08) |
| INTmed | .001 (-.008, .012) | .000 (-.019, .017) | 1.17 (.900, 1.56) |
| **LSES 🡪 Menopause Age 🡪 Y** |  |  |  |
| TE | .268 (.200, .335) | .134 (.005, .262) | .509 (.332, .780) |
| PNDE | .263 (.195, .330) | .133 (.004, .259) | .510 (.333, .781) |
| TNIE | .005 (-.002, .012) | .001 (-.005, .006) | .997 (.968, 1.03) |
| PNIE | .003 (-.006, .012) | .001 (-.006, .009 | 1.00 (.977, 1.04) |
| INTmed | .002 (-.004, .007) | .000 (-.007, .007) | .965 (.902, 1.03) |
| **LSES 🡪 Reproductive Span 🡪 Y** |  |  |  |
| TE | .269 (.201, .336) | .134 (.005, .262) | .500 (.325, .769) |
| PNDE | .260 (.198, .327) | .133 (.004, .259) | .504 (.329, .771) |
| TNIE | .005 (-.002, .012) | .001 (-.007, .009) | .992 (.948, 1.04) |
| PNIE | .002 (-.002, .010) | .001 (-.011, .013) | 1.01 (.970, 1.06) |
| INTmed | .002 (-.003, .013) | .000 (-.012, .011) | .957 (.896, 1.02) |
| **LSES 🡪 Breastfed 🡪 Y** |  |  |  |
| TE | .265 (.198, .333) | .129 (.002, .254) | .531 (.343, .825) |
| PNDE | .262 (.195, .330) | .124 (-.003, .250) | .525 (.339, .811) |
| TNIE | .003 (-.003, .009) | .004 (-.012, .024) | 1.01 (.971, 1.06) |
| PNIE | .000 (-.005, .005) | .003 (-.011, .022) | 1.01 (.980, 1.05) |
| INTmed | .003 (-.004, .010) | .000 (-.009, .007) | 1.02 (.723, 1.43) |
| **LSES 🡪 HRT Use 🡪 Y** |  |  |  |
| TE | .263 (.196, .330) | .138 (.010, .265) | .479 (.309, .745) |
| PNDE | .259 (.192, .326) | .132 (.003, .259) | .510 (.333, .784) |
| TNIE | .004 (-.002, .009) | .006 (.000, .012) | .930 (.850, 1.01) |
| PNIE | .003 (-.007, .014) | .001 (-.006, .010) | .987 (.944, 1.03) |
| INTmed | .000 (-.012, .012) | .005 (-.002, .015) | .940 (.870, 1.01) |
|  | **Baseline memory** | **Memory decline** | **Dementia Risk** |
| **Black Women** | b (95% CI) | b (95% CI) | HR (95% CI) |
| **LSES 🡪 EEI 🡪 Y** |  |  |  |
| TE | .263 (.209, .320) | .095 (.003, .187) | .479 (.365, .629) |
| PNDE | .261 (.205, .317) | .092 (.001, .187) | .480 (.367, .628) |
| TNIE | .002 (-.002, .006) | .003 (-.001, .009) | .998 (.959, 1.04) |
| PNIE | .002 (-.001, .008) | .001 (-.011, .008) | .995 (.975, 1.02) |
| INTmed | .000 (-.005, .006) | .002 (-.006, .009) | 1.02 (.987, 1.05) |
| **LSES 🡪 Menarche Age 🡪 Y** |  |  |  |
| TE | .252 (.202, .303) | .103 (.006, .199) | .472 (.359, .620) |
| PNDE | .246 (.195, .296) | .100 (.005, .196) | .488 (.373, .639) |
| TNIE | .009 (.001, .017) | .007 (-.003, .017) | .933 (.867, 1.00) |
| PNIE | .006 (.000, .016) | .002 (-.008, .015) | .959 (.921, .999) |
| INTmed | .003 (-.003, .012) | .003 (-.003, .012) | .987 (.964, 1.01) |
| **LSES 🡪 Menopause Age 🡪 Y** |  |  |  |
| TE | .263 (.208, .319) | .089 (.013, .165) | .477 (.364, .625) |
| PNDE | .263 (.208, .319) | .088 (.012, .164) | .477 (.364, .624) |
| TNIE | .000 (-.001, .001) | .001(-.005, .006) | 1.00 (.990, 1.01) |
| PNIE | .000 (-.001, .002) | .000 (-.003, .002) | .999 (.995, 1.01) |
| INTmed | .000 (-.001, .001) | .001 (-.004, .005) | 1.02 (.987, 1.05) |
| **LSES 🡪 Reproductive Span 🡪 Y** |  |  |  |
| TE | .264 (.207, .320) | .089 (.013, .165) | .477 (.364, .625) |
| PNDE | .263 (.207, .319) | .087 (.011, .162) | .477 (.365, .625) |
| TNIE | .001 (-.002, .003) | .001 (-.004, .007) | .999 (.984, 1.02) |
| PNIE | .001 (-.002, .003) | .000 (-.004, .003) | .998 (.988, 1.01) |
| INTmed | .000 (-.002, .002) | .001 (-.004, .007) | 1.01 (.985, 1.05) |
| **LSES 🡪 Breastfed 🡪 Y** |  |  |  |
| TE | .260 (.204, .317) | .105 (.002, .205) | .477 (.362, .630) |
| PNDE | .260 (.203, .316) | .104 (.001, .202) | .487 (.363, .631) |
| TNIE | .000 (-.002, .003) | .001 (-.007, .013) | .997 (.975, 1.02 |
| PNIE | .000 (-.00, .004) | .000 (-.007, .006) | .998 (.990, 1.01) |
| INTmed | .000 (-.003, .005) | .001 (-.004, .009) | .998 (.989, 1.01) |
| **LSES 🡪 HRT Use 🡪 Y** |  |  |  |
| TE | .261 (.206, .317) | .097 (.001, .190) | .480 (.367, .628) |
| PNDE | .252 (.196, .309) | .093 (-.004, .187) | .477 (.364, .625) |
| TNIE | .009 (.001, .018) | .003 (-.005, .011) | 1.00 (.981, 1.04) |
| PNIE | .006 (.000, .017) | .002 (-.008, .015) | 1.00 (.974, 1.03) |
| INTmed | .003 (-.004, .012) | .001 (-.011, .013) | .985 (.960, 1.01) |
|  | **Baseline memory** | **Memory decline** | **Dementia Risk** |
| **Latinx Women** | b (95% CI) | b (95% CI) | HR (95% CI) |
| **LSES 🡪 EEI 🡪 Y** |  |  |  |
| TE | .233 (.190, .278) | .089 (.010, .162) | .615 (.488, .773) |
| PNDE | .227 (.185, .269) | .083 (.004, .156) | .637 (.527, .771) |
| TNIE | .007 (-.004, .016) | .006 (-.005, .028) | .964 (.862, 1.08) |
| PNIE | .004 (-.003, .012) | .005 (-.005, .016) | .968 (.914, 1.02) |
| INTmed | .002 (-.005, .011) | .001 (-.016, .022) | .989 (.966, 1.01) |
| **LSES 🡪 Menarche Age 🡪 Y** |  |  |  |
| TE | .225 (.182, .268) | .085 (.006, .158) | .643 (.522, .792) |
| PNDE | .223 (.180, .266) | .079 (-.001, .152) | .641 (.530, .776) |
| TNIE | .002 (-.002, .006) | .006 (-.013, .029) | 1.00 (.939, 1.07) |
| PNIE | .000 (-.006, .006) | .004 (-.006, .016) | .990 (.957, 1.02) |
| INTmed | .002 (-.004, .010) | .002 (-.010, .014) | .957 (.878, 1.04) |
| **LSES 🡪 Menopause Age 🡪 Y** |  |  |  |
| TE | .234 (.192, .277) | .085 (.006, .158) | .617 (.512, .745) |
| PNDE | .231 (.189, .273) | .084 (.005, .157) | .619 (.514, .747) |
| TNIE | .003 (-.002, .008) | .001 (-.007, .013) | .996 (.979, 1.01) |
| PNIE | .001 (-.001, .005) | .001 (-.003, .005) | .998 (.988, 1.01) |
| INTmed | .001 (-.001, .006) | .000 (-.004, .006) | .989 (.964, 1.02) |
| **LSES 🡪 Reproductive Span 🡪 Y** |  |  |  |
| TE | .235 (.193, .278) | .085 (.006, .158) | .619 (.511, .749) |
| PNDE | .230 (.188, .272) | .084 (.005, .157) | .623 (.517, .751) |
| TNIE | .005 (-.001, .011) | .001 (-.003, .007) | .993 (.963, 1.03) |
| PNIE | .002 (-.001, .008) | .001 (-.006, .010) | .994 (.978, 1.01) |
| INTmed | .002 (-.001, .008) | .000 (-.010, .010) | .995 (.969, 1.02) |
| **LSES 🡪 Breastfed 🡪 Y** |  |  |  |
| TE | .221 (.177, .264) | .082 (.003, .155) | .655 (.524, .818) |
| PNDE | .221 (.177, .264) | .082 (.003, .155) | .654 (.535, .799) |
| TNIE | .000 (-.004, .004) | .000 (-.011, .019) | 1.00 (.941, 1.06) |
| PNIE | .000 (-.005, .004) | .000 (-.009, .008) | .998 (.962, 1.04) |
| INTmed | .000 (-.005, .003) | .001 (-.004, .012) | 1.01 (.951, 1.07) |
| **LSES 🡪 HRT Use 🡪 Y** |  |  |  |
| TE | .230 (.188, .273) | .085 (.006, .158) | .624 (.428, .911) |
| PNDE | .227 (.185, .270) | .079 (-.002, .150) | .740 (.431, 1.27) |
| TNIE | .003 (-.004, .010) | .007 (.001, .018) | .944 (.878, 1.01) |
| PNIE | .002 (-.004, .008) | .005 (.000, .010) | .927 (.837, 1.03) |
| INTmed | .000 (-.007, .005) | .002 (-.001, .005) | .809 (.646, 1.01) |

LSES = Lifecourse SES; TE = Total Effect; PNDE = the portion of the TE that is not mediated through the EEI; TNIE = portion of the TE transmitted through the EEI; PNIE = the indirect effect solely due to the mediated pathway; INTmed = the portion of the TNIE that results from the exposure-mediator interaction.
